# Genetic susceptibility for cow’s milk allergy in Dutch children: the start of the allergic march?

**DOI:** 10.1186/s13601-016-0096-9

**Published:** 2016-03-03

**Authors:** Peter Henneman, Nicole C. M. Petrus, Andrea Venema, Femke van Sinderen, Karin van der Lip, Raoul C. Hennekam, Marcel Mannens, Aline B. Sprikkelman

**Affiliations:** 1 Department Clinical Genetics, DNA-diagnostics laboratory, Amsterdam Medical Center, Amsterdam, The Netherlands; 2 Department of Paediatric Respiratory Medicine and Allergy, Emma Children’s Hospital, Amsterdam Medical Center, Amsterdam, The Netherlands

**Keywords:** Epigenetics, Food allergy, Gender differences, Cow’s milk allergy, Genetic susceptibility

## Abstract

**Background:**

Cow’s milk allergy (CMA) is the most common allergic disease in infancy. It is not clear, whether infants with CMA have an increased risk of developing other allergic diseases later in life, the so-called “allergic march”. We aimed to detect genetic associations of CMA using reported single nucleotide polymorphisms (SNP) in other allergic diseases and genetic mutations within the filaggrin (*FLG*) gene. Both to investigate possible causes of CMA, which also suggests an “allergic march”.

**Methods:**

Thirty children from the Dutch EuroPrevall birth cohort study with CMA in infancy and twenty-three healthy controls were studied. Six candidate SNPs were selected (minor allele frequency 10–50 % combined with a large effect) based on the literature. Thirteen *FLG* candidate mutations were selected spread over repeats 1, 3, 4, 5, 6, 7, 9 and 10 respectively.

**Results:**

We found two SNP’s, rs17616434 (P = 0.002) and rs2069772 (P = 0.038), significantly associated with CMA. One is located near the toll like receptor 6 (*TLR6)* gene, which functionally interacts with toll-like receptor 2, and is associated with an increased risk of other allergic diseases. One is located at the Interleukin 2 (*IL2*) locus. Twelve *FLG* amplicons were analyzed, but showed no significant enrichment. Nevertheless, we did observe more *FLG* mutations in the CMA-group compared to controls.

**Conclusion:**

We significantly associated two SNPs with CMA, suggesting that variation in the *TLR6* and *IL2* genes contribute to the expression of CMA. In addition, since *TLR6* and *IL2* were earlier associated with other later onset allergies, this also favours the “allergic march” hypothesis. We observed more *FLG* mutations in the CMA-group, albeit we found no statistical significant enrichment of *FLG* mutations. Further studies are necessary to investigate the role of common variants and *FLG* or other skin barrier gene mutations in CMA.

**Electronic supplementary material:**

The online version of this article (doi:10.1186/s13601-016-0096-9) contains supplementary material, which is available to authorized users.

## Background

### Cow’s milk allergy

Cow’s milk allergy (CMA) is the most common food allergy in young children, although an accurate incidence is difficult to establish because of discrepancies between self-reported and proper diagnosed allergy [1–4]. CMA and other food allergies have a heterogeneous clinical presentation, the estimated heritability of food-specific IgE ranged from 0.15 (cow’s milk) to 0.35 (wheat) [5, 6]. Young children are likely to develop tolerance for cow’s milk protein within a few years. However, infants who suffered from CMA in their early childhood seem to have an increased risk to develop other allergic diseases like asthma later in life [6–12]. Accumulating evidence suggest involvement of gut-microbiota, maturation of the immune-system and epicutaneous allergen sensitization [6, 13, 14]. Pre- and postnatal environmental factors, parent-of-origin factor and stress are likely to be involved in the susceptibility and expression of allergy [15]. So far, a clear cause for CMA has not been found.

### Genetic component of food allergy

Genetic surveys on food allergy (FA) are to our knowledge still limited to candidate gene studies, and studies investigating CMA solely are not available. GWAS on other atopic diseases like asthma and eczema identified few candidate genes, as presented by Bonnelykke et al. However, these genes were only found in single FA allergy studies, as presented by Tan et al. [16–18]. Recently, it was shown that loss-of-function mutations of filaggrin (*FLG*) are a major risk factor for peanut food allergy [19]. *FLG* has already been described in studies on eczema [16, 20]. The *FLG* protein is an important skin barrier protein, but is not expressed in the gastrointestinal tract [13, 19]. Studies on the sensitizing mechanisms involved in FA, suggest that exposure to food antigens through the skin, i.e. epicutaneous, leads to allergic sensitization while (early) oral administration of food antigens just may prevent the onset of allergy [13]. An impaired skin barrier is however not essential for epicutaneous sensitization. It has been shown that high molecular weight antigens can be taken up by dendritic cells, which are present in hair follicles, which on turn can either initiate a sensitization or repression of immunological responses [13, 21]. During a sensitization period, different immunological processes may lead to the full expression of allergy.

To summarize, susceptibility factors among different types of allergies other than CMA are well investigated. It is known that CMA is an ultimate complex disease involving different immunological pathways and different environmental factors. Here we aim to discover new insight in the genetic susceptibility of CMA by means of: (1) Genetic association of six allergy associated common and high effect genetic variants identified by GWAS and (2) genetic association of rare but high effect genetic mutations in the filaggrin gene.

## Methods

### Dutch EuroPrevall Birth Cohort Study

In this study only children participating in the Dutch EuroPrevall Birth Cohort Study were investigated. The EuroPrevall study has been described in detail previously [3, 4, 22, 23]. In summary, children were included around birth and standardized measurements were performed by questionnaires. All children with symptoms suggestive of CMA underwent, among others a double blind placebo controlled food challenge (DBPCFC). Age-matched healthy control children were selected from the entire Dutch cohort. In both symptomatic and control children measurements where repeated annually. Controls had no symptoms suggestive of any food allergy nor suffered from atopic dermatitis. In all children undergoing blood drawing an attempt was made to obtain one full blood sample (EDTA KE 2.6 ml Monovette, Sarstedt BV, Etten-Leur, Netherlands) for DNA-isolation too. The Medical Ethics Committee of the Academic Medical Hospital (METC 06/005) approved the Dutch EuroPrevall Birth Cohort Study. Written informed consent, for both the study and genetic sampling, was obtained from both parents of each child, unless only one of them had parental rights.

### CMA diagnosis

DBPCFC is the gold standard for diagnosing CMA and the challenge of choice according to the study protocol [22–24]. All children suspected of CMA were challenged according the international gold standard. The DBPCFC procedure was described in detail previously [3]. DBPCFC was repeated annually, in children with CMA, until the child was tolerant for CMP [3, 22]. Tolerance was defined as previously a positive DBPCFC for CMA, but at DNA-sampling a negative DBPCFC and/or eating CMP without experiencing symptoms.

### Samples selected for genetic analysis

DNA samples of 20 children with proven CMA were selected, based on the longest possible time between the age of DNA-sampling and the age of CMP-tolerance. Since DNA is generally assumed to be stable throughout life we state that former CMA patients and active CMA patients represent both CMA cases. Also ten samples of children with proven CMA who developed CMP tolerance during the course of the study were selected, called former CMA children. Together these 30 samples are called the CMA-group. Control samples (N = 23), were selected from the group of control children and matched on age at DNA-sampling.

Patient characteristics were analysed with *t* test (Mann–Whitney U test when data was not normally distributed) for continuous parameters. Chi square test was used for categorical variables and Chi square test for trend for multiple categories categorical variables. All characteristics were analysed using SPSS version 20 (IBM SPSS Statistics for Windows, Armonk, NY).

### Genetic association analysis

GWAS-studies reporting on SNPs associated with sensitization and/or allergy were selected [25]. SNP Inclusion criteria were based on the minor allele frequency (MAF) of 20–50 % and an odds ratio (OR) >1.2 [26, 27]. Based on the literature and our prior stated selection criteria, six SNPs were selected for evaluation. A description of the SNPs with regard nearby located genes is described in Table 1. Linkage disequilibrium (LD) was used to identify the most plausible involved gene, using LD plots per SNP on SNAP Broad institute with the following values: R^2^ = 0.8, distance limit = 500, CEU population [28]. Target reference sequences were downloaded using the database ENSEMBL [29]. These reference sequences were subsequently submitted to the web tool Primer3 in order to obtain a primer set. Primers were M13 tail extended [30]. Primer sequences of all 6 SNPs are described in Additional file 1: Table S1. Final quality control of the primer set was performed using the web tool SNPCheck [31].

**Table 1:** Candidate SNPs

SNP	Chr	Genotype*	HWP	Nearby gene(s): function
*rs2155219* ^a^	11	G/G: 0,30 G/T: 0,43	0.15	***C11orf30***: Regulator, represses transcription, possibly via its interaction with a multiprotein chromatin remodeling complex that modifies the chromatin. ***LRRC32***: Associated with allergic rhinitis and rhinitis
*rs17616434* ^b^	4	C/C: 0,06 C/T: 0,31 T/T: 0,62	0.371	***TLR10***: Toll Like Receptor 10. Pathogen activation, activation innate immunity. ***TLR1***: Toll Like Receptor 1. Specifically recognizes diacylated and triacylated lipopeptide. ***TLR6***: Toll Like Receptor 6. This receptor functionally interacts with toll-like receptor 2. associated with an increased risk of asthma in some populations. ***FAM114A1***: May play a role in neuronal cell development
*rs6586513* ^a^	1	A/A: 0,51 A/C: 0,49	0.15	***CROCC***: Major structural component of the ciliary rootlet. Contributes to centrosome cohesion before mitosis. ***ATP13A2***: ATPase Type 13A2. Transports inorganic cations and other substrates. ***SDHB***: succinate dehydrogenase complex. Complex 2 of respiratory chain. ***MFAP2***: Microfibrillar-associated protein. Major antigen of elastin-associated microfibrils
*rs3860069* ^a^	4	A/A: 0,50 A/C: 0,45 C/C: 0,05	0.343	***TLR6***: Toll Like Receptor 6. This receptor functionally interacts with toll-like receptor 2. associated with an increased risk of asthma in some populations. ***TLR10***: Toll Like Receptor 10. Pathogen activation, activation innate immunity. ***TLR1***: Toll Like Receptor 1. Specifically recognizes diacylated and triacylated lipopeptide. ***FAM114A1***: May play a role in neuronal cell development
*rs6898653* ^a^	5	A/A: 0,59 A/G: 0,36 G/G: 0,05	1	***SEMA6A***: expression in developing neuronal tissue. Is required for proper development of the thalamocortical projection
*rs2069772* ^a^	4	A/A: 0,52 A/G: 0,38 G/G: 0,10	0.527	***IL2***: Interleukin 2. Secreted cytokine for proliferation of T and B lymphocytes. Produced by T cells. ***ADAD1***: binds to RNA. Plays a role in spermatogenesis. ***KIAA1109***: associated with celiac disease

*Chr* chromosome

* CEU genotype, NCBI database, *HWP* Hardy–Weinberg p value, NCBI database

^a^candidate SNP earlier reported by Ramasamy. A. 2011

^b^candidate SNP earlier reported by Bønnelykke K. 2013

The *FLG* gene involves at least ten highly homolog repeat sequences. The primer sequences or primer design for detection of *FLG* mutations was based on the report of Sandilands et al. [32]. In total we selected 13 *FLG* candidate mutations spread over repeats 1, 3, 4, 5, 6, 7, 9 and 10 respectively (Table 2) [32]. Mutations reported only in the Chinese or Japanese population (3321delA and S2554X) were not included in this study. Since for most *FLG* primers specificity is based on not more than three repeat specific bases, the highest possible annealing temperature was used in the PCR in order to obtain the required regional specificity. All mutations were analysed using PCR, followed by Sanger sequencing. *FLG* Primers sequences are described in Additional file 1: Table S2. SNPs and *FLG* mutations were Sanger sequenced using standard protocols (BigDye Terminator^®^ mix, Applied Biosystems) and analysed using an ABI3730^®^ of Applied Biosystems.

**Table 2:** Selected filaggrin mutations according Sandilands et al.

FLG mutation	Amplicon	Repeat	g. (H19)	c.	ATG	bp	p.	Ethnicity^b^
R501X	FLG-1	1	152285861	1501	2072	C>T	501	EUR/AM
2282del4	FLG-2	1	152285080	2282	2853	del4 (CAGT-2286)	761/762	EUR/AM
3702delG	FLG-3	3	152283660	3702	4273	delG	1234	IR
R1474X	FLG-4	4	152282942	4420	4991	C>T	1474	IR
5360delG	FLG-5	5	152282002	5360	5931	delG	1787	NL
6867delAG	FLG-6	6	152280495	6867	7438	delAG (AG-2268)	2289/2290	AUS
E2422X	FLG-7	7	152280098	7264	7835	G>T	2422	NL
7267delCA	FLG-7	7	152280095	7267	7838	delCA (CA-7268)	2423	NL
R2447X	FLG-7	7	152280023	7339	7910	C>G	2447	IR
S3247X	FLG-8	9	152277622	9740	10311	C>A	3247	IR
11029delCA	FLG-9	10	152276333	11029	11600	delCA (CA-11030)	3677	IR
11033del4	FLG-9	10	152276329	11033	11604	Del4(CAGT-11036)	3678/3679	NL
Q3683X	FLG-9	10	152276312	11050	11050	C>T	3684^a^	Ir

^a^Sandilands reported p. 3683. *FLG* filaggrin, *g* genomic position, *c* coding postion,* ATG* distance from transcription start site, *bp* basepair change(s),* p.* affected amino acid

^b^Ethnic group were mutations were first reported, adapted from Sandilands et al. EUR (European), AM (American.), IR (Irish), AUS (Austrian), NL (Dutch)

Raw sequence data was analysed using CodonCode Aligner^®^ and Alamut^®^ software. Since all tested mutations in the *FLG* gene result in an absent or defective protein, we constructed a cumulative variant score per patient. This cumulative score included a score of zero risk alleles versus one or more risk alleles present in exon 3 repeats of *FLG*. The CMA-group was compared to controls. In population based genetic studies the Hardy–Weinberg equilibrium (HWE) is a golden standard for quality control of the analysed genotypes. Deviation of this equilibrium indicates genotyping errors or the presence of a certain genetic selection bias. Since we did analyze in this study a strongly selected sample of CMA patients, which obviously does not represent the general population, it would be inappropriate to apply the Hardy–Weinberg equilibrium as genotyping quality control in this study. All genetic statistical analyses were performed in SPSS (v20) using a Pearson’s Chi square test or Fisher’s exact test. P values <0.05 were considered significant.

## Results

Patient and control characteristics are shown in Table 3. Controls and children with CMA were well matched except for age at DNA-sampling. Children with CMA were significantly younger (P = 0.008) compared to controls. Our CMA group consists of both IgE as well as non-IgE mediated CMA, with slightly more IgE positive children (specific IgE >0.35 kU/L, P = 0.044) in the CMA group compared to controls. Comparison of the former CMA group, showed no significant difference in age at DNA-sampling (P = 0.71) nor IgE status (P = 0.49).

**Table 3:** Characteristics of Dutch allergy study population; cow’s milk allergy (CMA), former CMA and controls

	CMA	Control	P
N (♀)	20 (8)	20 (10)	*NS*
Age diagnosis CMA ± SD^a^	6.5 ± 2.5	*NA*	*NA*
Age at Sampling ± SD^a^	11.8 ± 4.9	17.2 ± 7.1	0.008
N of children with CMP specific sIgE >0.35 kU/L^b^	7	1	0.044
Mean CMP specific sIgE value ± SD (kU/L)	1.15 ± 3.6	0.16 ± 0.64	*NS*
Range IgE value (kU/L)	16.4	2.9	*NA*
	Former CMA	Control	P
N (♀)	10 (0)	13 (0)	*NA*
Age diagnosis CMA ± SD^a^	5.8 ± 2.2	*NA*	*NA*
Age at Sampling ± SD^a^	18.0 ± 5.6	17.2 ± 4.3	*NS*
Age at Tolerance ± SD^a^	17.2 ± 3.9	*NA*	*NA*
N sIgE >0.35 kU/L^b^	*0*	*0*	*NA*

*CMA* cow’s milk allergy, *N* number, *CMP* cow’s milk protein, *SD* standard deviation, *NA* not applicable, *NS* not significant, *sIgE* serum specific IgE

^a^age in months ANOVA

^b^Fisher’s exact test, 2-sided

### Genetic contribution of common variants to CMA

For two SNPs we observed significant enrichment of risk genotypes in the CMA-group compared to controls, illustrated in Table 4. For rs17616434, located on chromosome 4; *TLR10/1/6* and *FAM114A1* locus, C/C risk alleles were absent in the control group while 9 CMA/former CMA subjects showed this genotype (P = 0.002). The rs17616434 C/T and T/T genotypes were both enriched in the controls. For rs2069772 located on chromosome 4 as well, the T/T genotype was enriched in the CMA-group compared to the controls (80 vs. 46.7 % respectively), while the heterozygotes (C/T) and common genotype (C/C) was enriched in the controls (P = 0.038). Analysis based on the genotype frequencies within the CMA-group vs. controls was insignificant associated (P > 0.05) for rs2155219 (*LRRC32* locus), rs6586513 (*CROCC/ATP13A2/SDHB/MFAP2* locus), rs3860069 (*TLR6/10/1* and *FAM114A1* locus) and rs6898653 (*SEMA6A* locus).

**Table 4:** Comparison of SNPs between cow’s milk allergy (CMA), former CMA and controls

SNP	Group	Genotype details				P value
rs2155219		GG	GT	TT	Total	0.548
	CMA + former CMA	6 (24 %)	11 (44 %)	8 (32 %)	25 (100 %)	
	Control	7 (23.3 %)	17 (56.7 %)	6 (20 %)	30 (100 %)	
rs17616434		CC	CT	TT	Total	0.002
	CMA + former CMA	9 (36 %)	5 (20 %)	11 (44 %)	25 (100 %)	
	Control	0 (0 %)	10 (33.3 %)	20 (66.7 %)	30 (100 %)	
rs6586513		AC	AA		Total	0.456
	CMA + former CMA	12 (48 %)	13 (52 %)		25 (100 %)	
	Control	11 (37.9 %)	18 (62.1 %)		29 (100 %)	
rs3860069		CC	AC	AA	Total	0.336
	CMA + former CMA	4 (16 %)	8 (32 %)	13 (52 %)	25 (100 %)	
	Control	2 (6.7 %)	7 (23.3 %)	21 (70 %)	30 (100 %)	
rs6898653		GG	AG	AA	Total	0.951
	CMA + former CMA	1 (4 %)	13 (52 %)	11 (44 %)	25 (100 %)	
	Control	1 (3.4 %)	14 (48.3 %)	14 (48.3 %)	29 (100 %)	
rs2069772		CC	CT	TT	Total	0.038
	CMA + former CMA	1 (4 %)	4 (16 %)	20 (80 %)	25 (100 %)	
	Control	2 (6.7 %)	14 (46.7 %)	14 (46.7 %)	30 (100 %)	

*CMA* cow’s milk allergy

### Genetic contribution of rare FLG mutations to CMA

The investigated FLG-mutations were covered by 9 amplicons of which we successfully optimized PCR conditions for 8 amplicons. The 4th amplicon, covering the R1474X mutation in *FLG* repeat 4, did not show consistent results and was therefore excluded from analysis (data not shown). The latter mutation is extremely rare and was only reported in the Irish population [32].

**Fig. 1:**
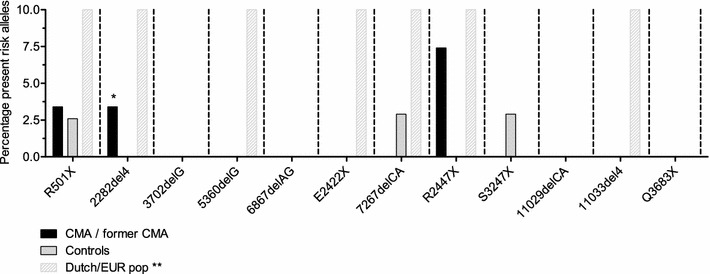
Enrichment analysis of filaggrin risk alleles in cow’s milk allergy patients vs. controls. Percent present risk alleles: total number of alleles/number of risk alleles per group [cow’s milk allergy (CMA) patients, former CMA patients and controls]. Dutch/EUR* pop column* indicates for every FLG mutation the discovery population according Sandilands et al. only and *does not* represent an actual percentage

**Fig. 2:**
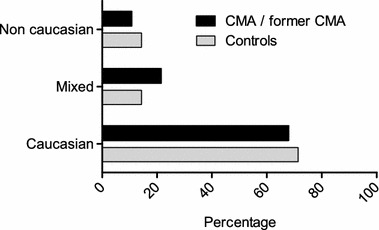
Ethnicity distribution among cow’s milk allergy (CMA), former CMA patients and controls Caucasian: both parents are of Caucasian background. Mixed: one parent is of Caucasian and one parent of non-Caucasian background. Non-Caucasian: both parents are of non-Caucasian background

**Fig. 3:**
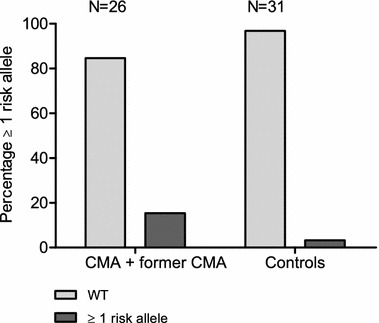
Enrichment analysis of filaggrin risk alleles in cow’s milk allergy patients vs. controls Percentage ≥1 risk alleles represents a cumulative score of all filaggrin (FLG) mutations within cow’s milk allergy (CMA) + former CMA and control groups. *WT* Wild type

Figure 1 illustrates the percentage of present risk alleles per group. According to the report of Sandilands et al. [32] some mutations were, so far, only observed in European and/or Dutch populations annotated as dashed bars in this figure. One sample, homozygote for the 2282del4 mutation involved a CMA patient. Geographic origin/ethnicity are known confounders in genetic surveys. Therefore, we evaluated the ethnic distribution (Fig. 2) and observed no difference between the two groups. All *FLG* mutations are rare, and in combination with a relative small sample, this implies extremely low statistical power. In order to diminish this power problem we counted per sample the number of present mutations yielding a cumulative *FLG* risk allele score. Samples who carried 1 or more risk alleles were annotated as 1. Figure 3 illustrates the scores in the CMA-group and controls. Although insignificant (Chi square test), more risk alleles carriers were present in the CMA-group compared to controls.

## Discussion

In this study we observed association of two earlier reported non-CMA allergies and/or allergy sensitization associated SNPs with CMA. Although insignificant, we observed more *FLG* mutations in the CMA-group compared to controls which suggest that a role of the *FLG*-gene in CMA cannot be excluded. Since our candidates were earlier reported to be associated with other types of late(r) onset allergic diseases, our data suggest not only that rs17616434, rs2069772 and *FLG* mutations are involved in CMA, but also suggest that these genetic variations might contribute to the so-called “allergic march”.

### Genetic analysis

We analysed six SNPs which have been described previously in relation to allergic diseases [26, 27]. Out of these six, two SNPs showed significantly different genotypic distribution between CMA and controls. First, rs17616434 (P = 0.002) was associated with CMA and is located near a cluster of toll like receptor (*TLR1, 6, 10*) genes, which has earlier been associated with allergic disease [33]. Secondly, rs2069772 (P = 0.038), was earlier described to be associated with allergic rhinitis, and is located near the *IL2* and *KIAA1109* genes [27]. *IL2* is known to be involved in cytokine secretion that stimulates proliferation of B and T -cells. The other gene, *KIAA1109*, is known to be involved in celiac disease, a disease characterized by a strong immunological response to food proteins (gluten), found in wheat, rye and barley [34]. Moreover, the *KIAA1109/Tenr/IL2/IL21* locus was also earlier associated with another immunological disorder, namely rheumatoid arthritis [35]. Therefore, both *IL2* and *KIAA1109* are good candidates to be involved in CMA. Earlier reports involving allergic diseases have shown that a defective *FLG*-gene was involved, albeit the prevalence of these mutations is ethnicity specific [32]. In our cohort we constructed a cumulative score of *FLG*-mutations. Although, the absolute number of mutations was higher in the CMA-group this difference was not statistical significant. The presence of ethnic specific mutations in our sample of mainly Dutch children was in concordance with the report of Sandilands et al., with exception of S3247X mutation, which was reported only in the Irish population [32]. Since *FLG* mutations are rare and our study sample is small we cannot state that *FLG* is not involved in CMA. To our knowledge, *FLG* has not been studied in CMA before. Therefore, studies involving a larger sample size are necessary to conclusively rule out or rule in the involvement of *FLG* in CMA.

### Allergic march

The allergic march hypothesize that children who suffered from food allergy or atopic dermatitis in early childhood have an increased risk of developing other allergic diseases, e.g. asthma and allergic rhinitis, in later in life. However, evidence for the existence of the allergic march is still very limited [6–12]. Recent reports describe pathways involved in allergic diseases and implied common genetic variation behind these affected pathways, among others, defects involving the skin barrier. The filaggrin protein is involved in maintaining a healthy skin barrier [18, 36]. Although in our study rare *FLG* mutations were not significantly enriched in CMA patients, we did observe more *FLG* mutations in our CMA group. The function of regulatory T-cells and the Th2 responses have been previously reported in relation to allergic diseases as well. These pathways have been associated with several other allergic diseases, which might favour the allergic march hypothesis, since the onset of these different types of allergies seem to manifest at specific ages [18, 36]. In our small sample study, we found a significant associated locus located close to the *IL2* gene which is involved in the Th2 response. Both our observations on *FLG* mutations and the *IL2* locus favour the allergic march hypothesis but have to be confirmed in a prospective study design.

### Strength and weaknesses of the study

The main strength of the studied samples is that CMA is diagnosed according to the current available, internationally recommended, gold standard [24]. Furthermore, clinical data are well documented due to the setup of the study with regular questionnaires [22]. Unfortunately, it was not possible to obtain DNA-samples in all children. Furthermore, the amount of blood drawing for research purposes in infants is in the Netherlands limited to 2.5 ml. These are both limiting the sample size of this study. This resulted in missing values for statistical analysis, however there were no significant differences in distribution between valid and missing cases between CMA-infants and controls (data not shown). Since this is only a very small sample size study in a very heterogeneous disease, further studies in larger cohorts are necessary. Obviously, many more genetic and environmental factors are involved in the development of CMA. With respect to an epidemiological approach unravelling mechanisms involved in CMA, large cohorts are essential. On the other hand, using well characterized small cohorts or even single cases might be essential to elucidate distinct mechanisms, that underlie at the basis of the complex character of CMA.

## Conclusion

Current studies indicates that genetic variation of *TLR6* and *IL2*, which were earlier reported to be associated with non-CMA allergies and/or allergy sensitization, contribute to the expression of CMA in young children. In addition, this favours the “allergic march” hypothesis. Furthermore, we cannot exclude a possible role for *FLG*-mutations being involved in CMA or the sensitization process prior to the establishment of CMA. Follow-up studies are necessary before definite conclusions about a link between early onset CMA and expression of later onset other allergic diseases can be drawn.

## Additional file

**Additional file 1.** Primer sequences. **a** Primer sequences candidate SNPs, **b** Primer sequences candidate Filaggrin (FLG) mutations.

## Abbreviations

CMA: cow’s milk allergy
CMP: cow’s milk protein
DBPCFC: double blind placebo controlled food challenge
DNA: deoxyribonucleic acid
FA: food allergy
*FLG*: filaggrin
GWAS: genome wide association studies
HWE: Hardy–Weinberg Equilibrium
LD: linkage disequilibrium
MAF: minor allele frequency
OR: odds ratio
SNP: single nucleotide polymorphism
