# Supplementary material for: Genetic susceptibility for cow’s milk allergy in Dutch children: the start of the allergic march?
Source: Clin Transl Allergy. 2016 Mar 3;6:7. doi: 10.1186/s13601-016-0096-9 (PMC4776421; doi:10.1186/s13601-016-0096-9)
Supplement: Supplementary file 1 — Additional file 1. Primer sequences. a Primer sequences candidate SNPs, b Primer sequences candidate Filaggrin (FLG) mutations. [file 13601_2016_96_MOESM1_ESM.doc]

**Table S1: Primer sequences candidate SNPs**

| **SNP** | **Forward** | **Reverse** |
| --- | --- | --- |
| rs2155219 | TGACTCATCATTTTGCAATGG | TGTGCTACATGGTAGAAATAATTTGA |
| rs17616434 | TGCCAAGGAATTCCCTATTCT | AAATATACGGCTGGCACCAC |
| rs6586513 | TCGGCTGTTAACCGAAAGAT | AGCCGGTTAAGAATCTGGTC |
| rs3860069 | CAGAGATAAGATTGATAATTTTGGCTGG | CAGCAGAGAAGTTTAGGTTTACTATCAA |
| rs6898653 | CAACCCCAAACCAACTCTTC | TCACTGGGCTTCTTTTGTCC |
| rs2069772 | CACATGAATGTTGTTTCAGATCC | CAAATGAGGAAAATCTCAGCTTC |

**Table S2: Primer sequences candidate Filaggrin (*FLG*) mutations**

| **FLG amplicon** | **Forward** | **Reverse** |
| --- | --- | --- |
| FLG-1 | CACTCATGAACAGCCTGACTC | GCCTGGAGCTGTCTCGTG |
| FLG-2 | ACAGTGGACACCGAGGGT | GCTCTGCTGATGGTGACCA |
| FLG-3 | CAAGCAGACAAACTCGTAAGGA | CCGATGATTGTTCCTGTCCC |
| *FLG-4* | *ACACGGACAGACTGCACC* | *TGGTGTGGCTGTGATGGTAC* |
| FLG-5 | GACAAGATTCATCTGTAGTCG | CCTGCTCGTGGCGGGATCT |
| FLG-6 | AGAGGCGGTCTGGGTCTGCG | CCAGAGGAATTCTCTGCATGAT |
| FLG-7 | CCACACGTGGCCGGTCAGCA | GTCCTGACCCTCTTGGGACGT |
| FLG-8 | CTAGACACTCACAGGCAGTC | CCTGATTGTCTGGAGCGGT |
| FLG-9 | GTCAGAGACAGTGGACACCGA | TACTGGAGGAAGACAAGGATC |
